# Editorial: Biomechanics, sensing and bio-inspired control in rehabilitation and wearable robotics

**DOI:** 10.3389/fbioe.2024.1487075

**Published:** 2024-10-23

**Authors:** Mingxiang Luo, Xinyu Wu, Ningbo Yu, Keyi Wang, Wujing Cao

**Affiliations:** ^1^ College of Engineering, Southern University of Science and Technology, Shenzhen, China; ^2^ Guangdong Provincial Key Lab of Robotics and Intelligent System, Shenzhen Institute of Advanced Technology, Chinese Academy of Sciences, Shenzhen, China; ^3^ College of Artificial Intelligence, Nankai University, Tianjin, China; ^4^ College of Mechanical and Electrical Engineering, Harbin Engineering University, Harbin, China

**Keywords:** exoskeleton, control, biomechanics, signal, rehabilitation

## Introduction

The integration of biomechanics, sensing technology, and bio-inspired control is transforming rehabilitation and wearable robotics by enhancing human mobility and recovery. Biomechanics informs the design of systems that replicate or support natural movement, while advanced sensors monitor physiological and biomechanical data in real time, enabling personalized assistance. Wearable robotics, such as exoskeletons and prosthetics, benefit from technologies like electromyography (EMG) and inertial measurement units (IMUs), which provide feedback for dynamic control adjustments. Bio-inspired control strategies further enhance these systems by mimicking the adaptability of biological systems, ensuring natural and efficient movement. This Research Topic documents recent advancements in these areas, emphasizing their role in improving mobility and rehabilitation outcomes for individuals with physical impairments. The 25 contributions can be organised into 6 main focus areas: (1) development and evaluation of wearable robotics; (2) control strategy studies; (3) signal and feature recognition; (4) biomechanical analysis; (5) literature review and statistical analysis; (6) rehabilitation training evaluation.

## Development and evaluation of wearable robotics

Xiang et al. conducted a study on back-support exoskeletons during manual material handling tasks, focusing on their biomechanical impact using Functional Data Analysis (FDA) and Functional ANOVA (FANOVA). The goal was to optimize exoskeleton design for safer reduction of lower back load. Participants performed tasks with and without the exoskeleton, while researchers collected data on lumbar load and trunk angle. FANOVA revealed that the exoskeleton significantly reduced lumbar load, particularly in lifting tasks, highlighting its effectiveness. The study also demonstrated FANOVA’s advantage in handling time-series data, providing valuable insights for designing better exoskeletal devices. Wang et al. developed a bedside cable-driven lower-limb rehabilitation robot for bedridden patients with neurological or limb disorders. This robot, based on sling exercise therapy, uses flexible cables to drive hip and knee motions at the bedside. A human-cable coupling controller dynamically adjusts the cable’s impedance in response to the patient’s joint impedance, stabilizing movement during rehabilitation. Experiments showed significant improvements in joint flexibility and stability, proving the robot’s effectiveness. Meng et al. designed a multi-degree-of-freedom, reconfigurable ankle rehabilitation robot with an adjustable workspace for post-stroke rehabilitation. The robot can be customized to meet individual needs, providing personalized and effective rehabilitation exercises. The study included finite element simulations to ensure structural integrity and safety, along with practical tests to validate its performance. Zha et al. developed a robot-assisted system for the reduction and rehabilitation of distal radius fractures, equipped with a robotic arm and integrated biplane radiographic imaging. This system enhances the accuracy and efficacy of closed reduction treatments by overcoming manual traction limitations and offering real-time radiographic assessment. Experiments confirmed that the system effectively achieves required traction forces and maintains wrist alignment, improving treatment protocols by making them less invasive, reducing recovery time, and minimizing radiation exposure. Liu et al. explored a knee exoskeleton driven by a series elastic actuator (SEA) for gait rehabilitation in stroke patients. They introduced a synergetic gait prediction model using an attention-based CNN-LSTM network to generate personalized gait trajectories, improving prediction accuracy and rehabilitation outcomes. Additionally, they proposed a compliant control scheme using an artificial potential field (APF) method to tune impedance parameters, ensuring safe and effective interaction between the robot and the patient. Jiao et al. developed a Reconfigurable Multi-Terrain Adaptive Casualty Transport Aid (RMTACTA) for industrial environments, enhancing pre-hospital casualty transport. The device uses a Watt II 6-bar linkage mechanism to transition between multiple modes, facilitating navigation across various terrains. A single remote rope controls the system, ensuring adaptability and ease of operation. A prototype verified the design’s functionality, demonstrating significant improvements in casualty transport efficiency and safety. Liu et al. presented a novel non-back-drivable clutch-based self-locking mechanism to improve stability in prosthetic joints. This mechanism allows precise positioning without changing the transmission ratio, which is critical for prosthetic wrists requiring reliable performance. The design minimizes friction during operation and ensures that the prosthetic limb remains fixed even during power failures, enhancing safety and comfort. The study included detailed mechanical design, kinematic analysis, and extensive testing, proving the mechanism’s effectiveness and durability.

## Control strategy study

Zhang et al. studied adaptive impedance control for an upper limb rehabilitation robot, focusing on dynamically adjusting training parameters based on patient status. Using a two-degree-of-freedom flexible drive joint and a forgetting factor recursive least squares method, they successfully estimated and optimized impedance parameters, significantly improving rehabilitation effectiveness by tailoring assistance to real-time patient needs. Tian et al. proposed a force/position-based velocity control (FPVC) strategy for a lower limb rehabilitation robot, enhancing trajectory tracking and patient participation. Their extensive experiments demonstrated that this approach improves interaction and rehabilitation outcomes. Li et al. introduced an “Orbit Energy” (OE) metric to enhance lower limb exoskeleton stability during standing. This metric helps select balance recovery strategies, such as adjusting ankle and hip torque, significantly improving balance and reducing muscle activation during disturbances. Liang et al. developed a multi-mode adaptive control strategy for lower limb rehabilitation robots, including robot-dominant, patient-dominant, and safety-stop modes. This strategy dynamically adjusts assistance based on patient abilities, improving rehabilitation outcomes and ensuring safety, as validated through simulations.

## Signal and feature recognition

Gong et al. explored multimodal fusion and human-robot interaction control in an intelligent robotic walker for gait rehabilitation, aiming to improve support and guidance for stroke patients. By integrating sensors like force sensors, joysticks, and depth-sensing cameras, the walker dynamically adjusts to the user’s motion intentions, enhancing walking assistance and potentially improving rehabilitation outcomes. Sarasola-Sanz et al. studied a hybrid brain-muscle-machine interface (hBMI) for stroke rehabilitation, involving six severely paralyzed patients. The hBMI, which combines EMG with brain signals to control an upper limb exoskeleton, showed significant improvements in arm function and neural engagement, demonstrating its potential for effective motor recovery. Feng et al. developed a method using surface electromyography (sEMG) signals to identify coordinated movement intentions in a multi-posture rehabilitation robot. By optimizing features with genetic algorithms, their model accurately recognized movement intentions, enhancing interactive rehabilitation training. Zhou et al. addressed the Sim2Real challenge in soft robotics by introducing the ImbalSim2Real scheme, which optimizes model transition from simulation to real-world data using techniques like discriminator-enhanced samples. Their approach improved bio-signal estimation in medical applications, particularly in soft robot-assisted rehabilitation. Yi et al. developed TGANet, a deep learning model that integrates an attention mechanism into VGG16 to improve the classification of tongue features in Traditional Chinese Medicine. TGANet outperformed traditional models in accuracy, precision, F1 score, and AUC, demonstrating its effectiveness in enhancing diagnostic accuracy and rehabilitation outcomes in TCM.

## Biomechanical analysis

Kang et al. studied the biomechanical impact of material anisotropy in 3D printed vertebral body implants for spinal reconstruction. They compared linear elastic isotropy and nonlinear anisotropy models using finite element analysis under various load conditions. Their findings show that the anisotropic model better represents the spinal system’s mechanical behavior, with lower stress levels and displacement, suggesting higher safety and stability in spinal reconstructions when anisotropic properties are considered. This research offers valuable insights for improving spinal implant design and clinical outcomes. Shakourisalim et al. conducted a comparative study on the biomechanical impact of manual material handling tasks using back support exoskeletons and assistive tools in both laboratory and real-world settings. They found significant differences in muscle activation between the two environments, highlighting the importance of real-world assessments for accurately evaluating the ergonomic benefits of exoskeletons. Despite these differences, ergonomic risk, measured by REBA scores, remained consistent across settings. This study underscores the need for field assessments to fully understand the impact of ergonomic interventions.

## Literature review and statistical analysis

Li et al. reviewed the use of extended reality (XR) technologies, including virtual reality (VR), augmented reality (AR), and mixed reality (MR), in training for myoelectric prostheses. They found that XR enhances training by providing immersive, interactive environments that increase user motivation and skill acquisition. However, challenges remain in translating virtual skills to real-world prosthesis control and improving training protocols. The authors suggest that XR holds promise for advancing prosthetic training and improving clinical outcomes. Zheng et al. examined the challenges in anthropomorphic motion planning for multi-degree-of-freedom robotic arms, focusing on creating humanoid robots with natural, human-like movements. They identified three key areas—motion redundancy, Research Topic, and coordination—as essential for improving robot interactions in various environments, including service, industrial, and healthcare settings. The research emphasizes integrating biomechanics, neurophysiology, and advanced computational models to mimic human movement effectively. Wen et al. conducted a bibliometric and visual analysis of research trends in robotic exoskeleton-assisted walking rehabilitation for stroke patients. Using data from the Web of Science Core Research Topic, they identified a rise in publication volume over the past decade, highlighting key research areas such as exoskeleton technology development, machine learning applications, and the impact on patient quality of life. This analysis offers insights into the current state and future directions of robotic exoskeleton research in stroke rehabilitation.

## Rehabilitation training evaluation

Hu et al. studied how walking speed affects gait stability using multi-scale entropy analysis and plantar pressure measurements. They found that slower walking speeds offer greater stability, particularly in elderly populations, providing insights for designing safer walking practices and rehabilitation strategies. Liu et al. evaluated motion compensation in post-stroke rehabilitation using muscle synergy indicators and surface electromyography. Their study, involving stroke patients and healthy subjects performing hand-cycling tasks, showed that synergy symmetry and fusion effectively measure motion compensation, suggesting ways to optimize rehabilitation strategies. Wang et al. developed a finite element model to assess the impact of various rehabilitation methods on urinary and defecation control in elderly men. Their study found that targeted exercises for the levator ani, external anal sphincter, and pelvic floor muscles were particularly effective, emphasizing the importance of personalized rehabilitation programs. Another study by Wang et al. examined the effectiveness of combining diaphragmatic breathing with limb coordination training for treating lower limb lymphedema after gynecologic cancer surgery. They found that combining these exercises with complex decongestive therapy significantly improved symptoms, reduced limb circumference, and alleviated anxiety and depression, suggesting enhanced rehabilitation outcomes for these patients.

## Summary

This Research Topic integrates the latest advancements in biomechanics, sensing technology, and bio-inspired control in the fields of rehabilitation and wearable robotics, demonstrating how these technologies can enhance human mobility and rehabilitation outcomes. The research findings are categorized into six main areas: development and evaluation of wearable robotics, control strategy studies, signal and feature recognition, biomechanical analysis, literature review and statistical analysis, and rehabilitation training evaluation. These studies not only expand our understanding of rehabilitation technologies but also provide new approaches for personalized rehabilitation interventions. Future research could delve deeper into several key questions: How can bio-inspired control be combined with real-time sensing data to achieve more precise personalized rehabilitation? How can signal recognition technology enhance devices “ability to perceive patients” intentions, thereby improving human-machine interaction? Additionally, with the application of virtual reality and mixed reality technologies in rehabilitation, exploring their potential to boost motivation and effectiveness in rehabilitation training is of great importance. These directions could not only deepen the understanding of current research findings but also provide strong support for the development of future rehabilitation devices and control strategies, paving the way for future research topics.

